# circFADS2 regulates lung cancer cells proliferation and invasion via acting as a sponge of miR-498

**DOI:** 10.1042/BSR20180570

**Published:** 2018-08-31

**Authors:** Fucheng Zhao, Yanru Han, Zhenzhou Liu, Zhenxia Zhao, Zhuoran Li, Kui Jia

**Affiliations:** 1 Department of Integrated Chinese and Western Medicine, The First Affiliated Hospital of Xinxiang Medical University, Weihui 453100, Henan, China; 2 Department of Oncology, The First Affiliated Hospital of Xinxiang Medical University, Weihui 453100, Henan, China

**Keywords:** circFADS2, invasion, lung cancer, miR-498, proliferation

## Abstract

CircRNAs could play critical functions in tumor progression. However, the expression and underlying mechanism of circRNAs in lung cancer progression remain poorly defined. In the present study, high-throughput microarray assay revealed that hsa_circRNA_100833 (identified as circFADS2) was markedly evaluated in lung cancer tissues, and it was further validated by qRT-PCR. High expression of circFADS2 was correlated with advanced TNM stage, lymph node metastasis, poor differentiation, and shorter overall survival of NSCLC patients. *In vitro* assays results showed that circFADS2 inhibition suppressed lung cancer cells proliferation and invasion ability. Bioinformatics analysis showed that miR-498 contained the complementary binding region of circFADS2, which was confirmed by Dual-luciferase reporter assay. In addition, the expression of miR-498 was down-regulated and negatively associated with circFADS2 expression in nonsmall cell lung cancer. Furthermore, rescue assays showed that miR-498 inhibitors abolished the effects of circFADS2 inhibition on lung cancer cells progression. Taken together, our findings indicated that circFADS2 was an effective tumor promoter in lung cancer progression, and its functions were performed by regulating the expression of miR-498. These data suggested that circFADS2 could act as a target for lung cancer treatment.

## Introduction

Lung cancer is the leading cause of cancer related death both in men and women worldwide with more than 80% of lung cancer being classified as nonsmall cell lung cancer (NSCLC) [1,2]. Despite the improvement of diagnostic approaches and targeted therapies, the 5-year overall survival rate remains as low as 15% [3,4]. Therefore, it is of great importance to uncover the molecular mechanism of cancer cells proliferation and metastasis in NSCLC to facilitate the developments of new drugs.

Circular RNAs (circRNAs) are considered as RNA molecules in loop structure generated from the aberrant splicing of the transcripts [5,6]. Increasing evidence showed that circRNAs could play important roles in many biological processes, such as cell proliferation, invasion, and differentiation [7,8]. It has also been reported that circRNAs have functions in a variety of tumors, including lung cancer. For example, Zong et al. showed that circRNA_102231 was increased and could act as a potential biomarker and therapeutic target for lung cancer patients [9]. Jiang et al. [10] indicated that circular RNA hsa_circ_0007385 could function as an oncogene in nonsmall cell lung cancer tumorigenesis. Wan et al. [11] indicated that circular RNA-ITCH suppressed lung cancer proliferation via inhibiting the Wnt/β-catenin pathway.

MicroRNAs (miRNAs) are a class of short noncoding RNAs of approximately 22 nucleotides in length and regulate gene expression by binding to the complementary sequence in the 3′-UTR region of target mRNAs [12]. Recent studies showed that circRNAs could act as miRNA sponges to modulate gene transcription in tumorigenesis. For example, Zeng et al. [13] showed that CircHIPK3 promoted colorectal cancer growth and metastasis by sponging miR-7. Huang et al. [14] revealed that silencing hsa_circ_0000977 suppressed pancreatic ductal adenocarcinoma progression by stimulating miR-874-3p and inhibiting PLK1 expression. Yang et al. [15] indicated that circ-ITCH inhibited bladder cancer progression by sponging miR-17/miR-224 and regulating p21, PTEN expression.

In the present study, our data suggested that the expression of circFADS2 was increased and associated with advanced TNM stage, lymph node metastasis, poor differentiation, and poor overall survival of NSCLC patients. circFADS2 inhibition suppressed lung cancer cells proliferation, migration, and invasion ability. Furthermore, our results showed that circFADS2 could act as miR-498 sponge to mediate the tumorigenicity.

## Materials and methods

### Patients and specimens

A total of 43 pairs of human NSCLC and adjacent nontumor tissues were collected at The First Affiliated Hospital of Xinxiang Medical University. The patients selected had not received any chemotherapies or radiotherapies before surgery. Tissue samples were immediately snap-frozen in liquid nitrogen. All human materials were obtained with written informed consent, and the present study was approved by the Institute Research Ethics Committee at The First Affiliated Hospital of Xinxiang Medical University. The clinical features of NSCLC patients are summarized in Table 1.

**Table 1:** Correlation between circFADS2 expression and clinicopathological features in NSCLC patients

Parameters	Group	Total	lncRNA circFADS2		*P*value
			Low	High	
Gender	Male	26	12	14	0.663
	Female	17	9	8	
Age (years)	<60	14	8	6	0.449
	≥60	29	13	16	
Tumor size (cm)	<3 cm	19	11	8	0.290
	≥3 cm	24	10	14	
Histology	Adenoma	20	8	12	0.280
	Squamous	23	13	10	
TNM stage	I	15	11	4	**0.019**
	II–III	28	10	18	
Lymph node metastasis	Absence	18	15	3	**0.000**
	Presence	25	6	19	
Differentiation	Well	16	12	4	**0.008**
	Moderate to Poor	27	9	18	

### Microarray analysis

Human circRNA expression analysis was performed on five pairs of NSCLC tissues and normal lung tissue. Sample preparation and microarray hybridization were performed according to Arraystar standard protocols (Rockville). circRNAs were enriched through removing linear RNAs with Rnase R (Epicentre). Amplified and labeled using Arraystar Super RNA Labeling Kit (Arraystar) and then scanned by the Agilent Scanner G2505C. The circRNA microarray process was performed by KangChen Biotech, Inc. (Shanghai, China).

### Quantitative real-time PCR

Total RNA was isolated from tissues and cell lines by using the TRIzol reagent (Invitrogen). The complementary DNA (cDNA) was carried out starting from 100 ng of total RNA. The relative expression of circRNA and miRNA were detected by using the SYBR® Green Master Mix (Takara) using a Step One Plus Real-Time PCR system (Applied Biosystems). circRNA was validated via PCR reaction using divergent and convergent primers. GAPDH was used as an endogenous control. The relative expression was calculated using 2^−ΔΔ*C*^_t_ methods. Primers were synthesized by GenePharma (Shanghai, China).

### Cell culture and transfection

Four human NSCLC cell lines (A549, SK-MES-1, H1299, and NCI-H1975) and normal human osteoplastic cell line (NHOst) were purchased from Cell Bank of the Chinese Academy of Science (Shanghai, China). All cell lines were maintained in Dulbecco modified Eagle medium (DMEM, Gibco) containing 10% fetal bovine serum (FBS, Gibco) and 100 U/ml penicillin and 100 μg/ml streptomycin at 37°C in a humidified incubator with 5% CO_2_.

The small interfering RNAs (si-circFADS2 and si-NC) were synthesized and purchased from GenePharma (Shanghai, China). MiR-498 inhibitors, miR-498 mimics, and the corresponding mimic/inhibitor negative control (NC) oligos were purchased from Invitrogen. Cells transfected with siRNAs or miR-498 inhibitors/mimics using Lipofectamine 2000 (Invitrogen) following the manufacturer’s protocol.

### Cell proliferation assay

Lung cancer cells were seeded in 96-well plates for 2 days. Cell Counting Kit-8 (CCK-8, Dojindo) was used to assess the cell proliferation. In general, 10 µl of CCK-8 solution was added to each plate and cells were incubated for 2 h. The cell viability was revealed by the absorbance which was measured at 450 nm. Proliferation rates were determined at 24, 48, 72, and 96 h after transfection.

### Wound healing assay

Cell migration ability was measured using wound healing assay. Cells were cultured on six-well plates. On the next day, a wound was made by scratching the central of each well with a 10 μl pipette. The culture medium was then changed with fresh medium containing 1% FBS. The first image of the scratch was captured. After 24 h, the second image of the scratch was acquired. The migration percentage was analyzed using Image Pro Plus.

### Transwell invasion assay

Cell invasion was evaluated using 24-well precoated with 100 µg Matrigel (Becton-Dickinson). Briefly, 1 × 10^5^ transfected cells were resuspended in 500 µl of serum-free medium and placed in the upper chamber with the lower chamber containing 500 µl of DMEM and 10% FBS. After 48 h, cells in the lower chamber were fixed with 4% paraformaldehyde in PBS for 10 min at room temperature and stained with 1% Crystal Violet for 30 min at room temperature. The invasive cells were counted under a microscope and the relative number was calculated.

### Dual-luciferase assay

For the luciferase reporter assay, the pGL3 plasmid encoding a luciferase reporter gene was purchased from Promega Corporation (Madison). Recombinant plasmid of pGL3-circFADS2-Wt and Mut was constructed. HEK-293T cells were cotransfected with miR-498 mimics or miR-NC, pGL3-circFADS2-Wt and Mut using Lipofectamine 2000 (Invitrogen). The pRL-TK vector was used as a normalization control. After transfection for 48 h at 37°C, cells were harvested and assayed with a Dual-Luciferase Reporter Assay system (Promega) according to the manufacturer’s protocol.

### Statistical analysis

All statistical analyses were performed using SPSS 18.0. The data were presented as means ± SD. Differences between two groups were analyzed by Student’s *t* test or one-way ANOVA. *P*<0.05 was considered statistically significant.

## Results

### CircRNA profiles in NSCLC tissues

**Figure 1:**
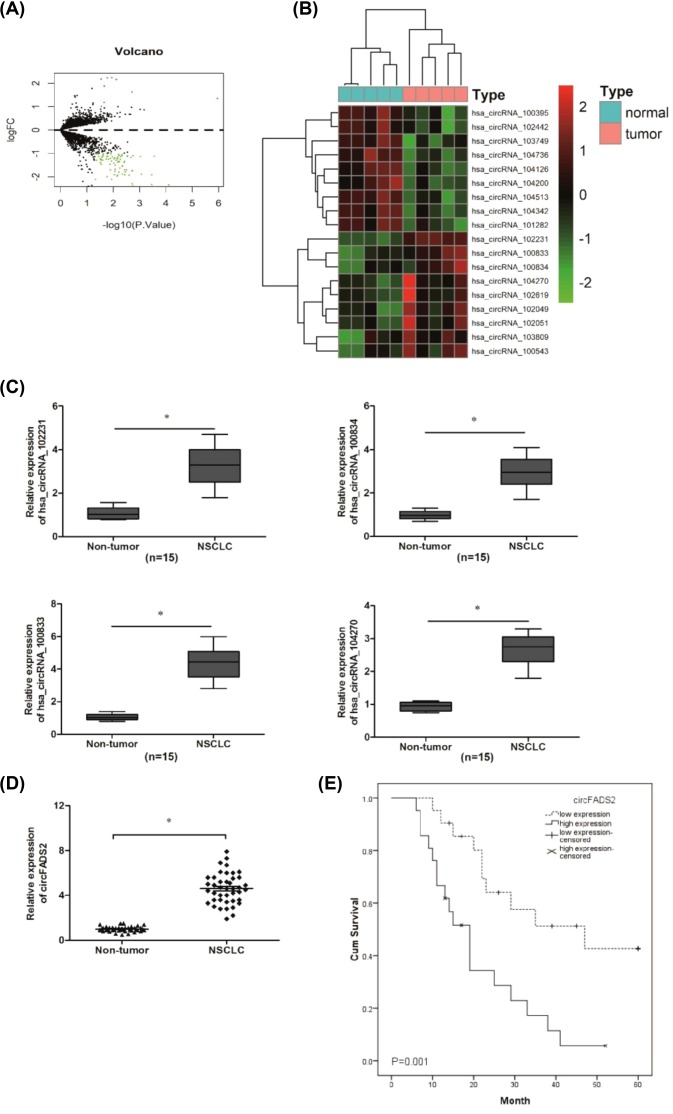
CircRNA expression profile in NSCLC tissues (**A**) Scatter-plot of circRNAs expression in NSCLC tissues and normal lung tissues. (**B**) Heat maps showed the top 9 up-regulated and down-regulated circRNAs in NSCLC tissues and normal lung tissues. (**C**) Expression of four indicated circRNAs listed in (**B**) were determined by qRT-PCR in 15 paired NSCLC tissues. (**D**) The expression of circFADS2 was detected by qRT-PCR in 43 paired NSCLC tissues. (**E**) Kaplan–Meier survival rate analysis based on circFADS2 expression in NSCLC patients; *P<0.05.

To identify specific circRNAs in NSCLC, five paired NSCLC tissues and nontumor tissues were subjected for circRNA microarray assay. The volcano plots revealed the variation of circRNA expression between NSCLC samples and nontumor tissue samples (Figure 1A). Through expression intensity sorting within NSCLC tissues and nontumor groups, the nine mostly up-regulated and down-regulated circRNAs in NSCLC were shown in Figure 1B. Next, we examined mostly four upregulated expression circRNAs in 15 paired NSCLC tissues. Results showed all those circRNAs were significantly up-regulated in NSCLC tissues, and we selected the most markedly up-regulated hsa_circRNA_100833 (gene symbol:FADS2; identified hsa_circRNA_100833 as circFADS2) for our further study (Figure 1C; *P*<0.05).

Furthermore, the expression levels of circFADS2 were further confirmed in another 43 paired NSCLC tissues. qRT-PCR results showed that the expression of circFADS2 was markedly increased in NSCLC tissues (Figure 1D; *P*<0.05). The median value of circFADS2 in NSCLC tissues was used as a cutoff value, and all patients were divided into two groups: high-circFADS2 expression group and low-circFADS2 expression group, correlations analysis showed that the high expression of circFADS2 was associated with advanced TNM stage, lymph node metastasis, and poor differentiation in NSCLC patients (Table 1; *P*<0.05). However, the expression of circFADS2 was not associated with other clinical features of NSCLC patients (Table 1; *P*>0.05). In addition, Kaplan–Meier analysis with log-rank test showed that NSCLC patients with high expression of circFADS2 had a shorter overall survival compared with patients with low expression of circFADS2 (Figure 1E; *P*<0.05). Those data indicated that circFADS2 could play a critical role in lung cancer progression.

### Knockdown of circFADS2 inhibits NSCLC cells proliferation and invasion

**Figure 2:**
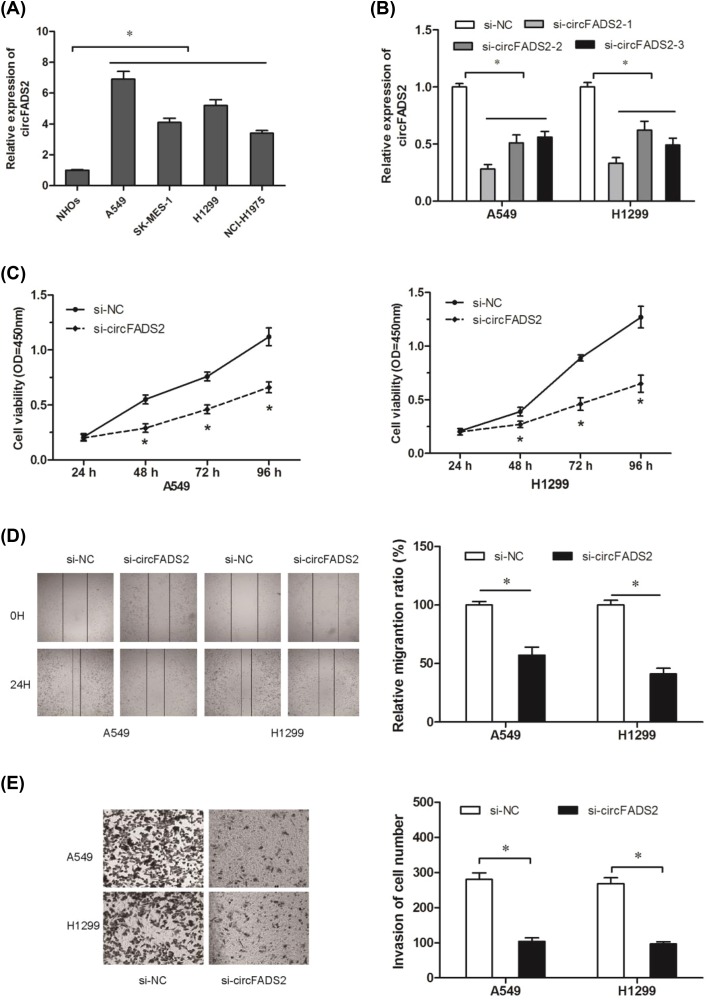
Decrease circFADS2 expression inhibited cell proliferation and invasion (**A**) The expression of circFADS2 in four lung cancer cell lines (A549, SK-MES-1, H1299, and NCI-H1975) and human osteoplastic cell line NHOst42 were explored by qRT-PCR. (**B**) The expression of circFADS2 was significantly decreased in cells transfected with si-circFADS2. (**C**) CCK-8 assay was used to determine the proliferation of A549 and H1299 cells transfected with si-circFADS2 or si-NC. (**D**) The migration of A549 and H1299 cells transfected with si-circFADS2 or si-NC was explored by wound healing assay. (**E**) The invasion of A549 and H1299 cells transfected with si-circFADS2 or si-NC was detected by transwell invasion assay; *P<0.05.

Next, we explored the expression of circFADS2 in four lung cancer cell lines (A549, H1299, SK-MES-1, and NCI-H1975). We found that circFADS2 expression was significantly up-regulated in lung cancer cells compared with human osteoplastic cell line NHOst (Figure 2A; *P*<0.05). Then, we transfected si-circFADS2 and si-NC into A549 and H1299 cells, qRT-PCR showed the expression of circFADS2 was markedly down-regulated in cells transfected with si-circFADS2 compared with si-NC group (Figure 2B; *P*<0.05). CCK-8 assay showed that circFADS2 knockdown markedly reduced lung cancer cells proliferation ability (Figure 2C; *P*<0.05). In addition, wound healing assay and transwell invasion assay showed that circFADS2 inhibition significantly reduced the migration and invasion ability of A549 and H1299 cells (Figure 2D,E; *P*<0.05). These data indicated that circFADS2 might act as an oncogenic circRNA in NSCLC tumorigenesis.

### MiR-498 acted as one of the target of circFADS2

**Figure 3:**
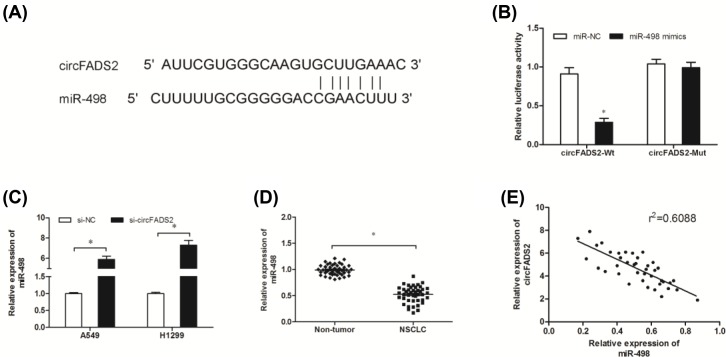
MiR-498 was a target of circFADS2 (**A**) The schematic showed putative binding sites for circFADS2 and miR-498. (**B**) Luciferase activity was detected in cells transduced with miR-498 mimics or miR-NC. (**C**) Si-circFADS2 inhibition up-regulated miR-498 expression in lung cancer cells. (**D** and **E**) The expression of miR-498 was markedly reduced and negatively associated with the expression of circFADS2 in NSCLC tissues;*P<0.05

Recent studies showed that circRNA could act as a miRNA “sponge” and inhibit miRNAs’ function [16]. Therefore, we hypothesized that circFADS2 could act as miRNA sponges to mediate lung cancer progression. Bioinformatics analysis showed that miR-498 shared complementary binding sites with circFADS2 (Figure 3A). Luciferase reporter assay revealed that luciferase activity of miR-498 binding with circFADS2-Wt was significantly reduced (Figure 3B; *P*<0.05). qRT-PCR showed that miR-498 expression was increased in NSCLC cells transfected with si-circFADS2 (Figure 3C; *P*<0.05). Furthermore, we determined the expression of miR-498 in NSCLC tissues, qRT-PCR showed that the expression of miR-498 was significantly decreased and negatively correlated with circFADS2 expression in NSCLC tissues (Figure 3D,E; *P*<0.05).

### MiR-498 inhibitors reversed the function of circFADS2

**Figure 4:**
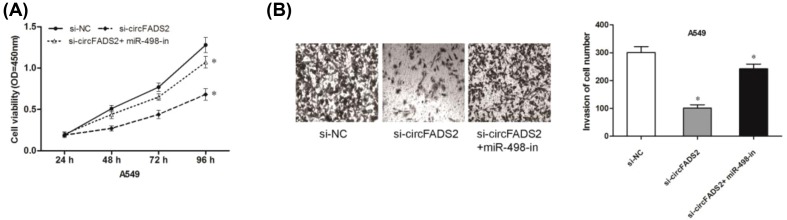
MiR-498 inhibition reversed the effects of si-circFADS2 on A549 cells (**A**) The proliferation of cells transfected with miR-498 inhibitors and si-circFADS2 was detected by CCK-8 assay. (**B**) The invasion of cells transfected with miR-498 inhibitors and si-circFADS2 was detected by transwell invasion assay; *P<0.05

Furthermore, we explored whether miR-498 inhibitors could abolish effects of circFADS2 inhibition on NSCLC progression. CCK-8 assay showed that miR-498 inhibitors significantly reversed the inhibition of cell proliferation induced by si-circFADS2 (Figure 4A; *P*<0.05). Moreover, transwell invasion assay showed that miR-498 inhibitors reversed the effects of circFADS2 suppression on lung cancer cells invasion (Figure 4B; *P*<0.05). Thus, those data suggested that circFADS2 could promote lung cancer tumorigenesis by regulating miR-498 expression.

## Discussion

Lung cancer is one of the most heterogeneous diseases whose underlying mechanisms are still unclear. Early diagnosis, radical surgery, and chemotherapy have improved prognosis of lung cancer patients; however, the mortality rates remain high [3]. With the development of high-throughput sequencing or next-generation sequencing, increasing numbers of new circRNAs have been discovered and identified in many diseases, including tumor progression [17,18]. For example, Zhou et al. [19] showed that circular RNA Atp9b could regulate the progression of osteoarthritis by targeting miR-138-5p. Wei et al. [20] revealed that circLMO7 could regulate myoblasts differentiation and survival by sponging miR-378a-3p. He et al. [21] found that circGFRA1 and GFRA1 could act as ceRNAs in triple negative breast cancer by regulating miR-34a. Thus, in the present study, we focus on the role of circRNAs in lung cancer tumorigenesis.

In the present study, the expression profiles of circRNAs were detected in NSCLC tissues using circRNA microarray analysis. Results showed that there are 9 circRNAs up-regulated and 79 circRNAs down-regulated with fold-changes >2.0 and *P*-values <0.05. From these candidate circRNAs, most markedly we choose the most up-regulated hsa_circRNA_100833 (identified as circFADS2) for our further study. Hsa_circRNA_100833 is located at chr11 and its associated-gene symbol is FADS2 (identified as circFADS2). We found that increased expression of circFADS2 was associated with advanced TNM stage, lymph node metastasis, poor differentiation, and poor overall survival in NSCLC patients. *In vitro* assays showed that circFADS2 suppression decreased A549 and H1299 cells’ proliferation and invasion ability. Those data indicated that circFADS2 could act as an oncogenic circRNA in lung cancer’s progression.

MicroRNAs (miRNAs) are a class of short noncoding RNAs and regulate gene expression by binding to the complementary sequence in the 3′-UTR region of target mRNAs [12]. More and more studies showed that circRNAs could act as miRNA sponges to modulate gene transcription in tumorigenesis [7]. Recent studies showed that miR-498 could play critical roles in tumor progression. For example, Cong et al. [22] revealed that decreased expression levels of miR-498 were associated with worse overall survival and poor prognosis in patients with ovarian cancer. Islam et al. [23] showed that miR-498 was reduced in esophageal squamous cell carcinoma and regulated tumor progression via regulating FOXO1/KLF6 transcriptional axis. Liu et al. [24] showed that miR-498 regulated FOXO3 expression and inhibited the proliferation of human ovarian cancer cells. Furthermore, Wang et al. [25] showed that miR-498 was down-regulated in nonsmall cell lung cancer and correlated with tumor progression.

In the present study, bioinformatics predicted a putative complementary region of circFADS2 and miR-498, which was confirmed by luciferase reporter assay. qRT-PCR showed that miR-498 expression was decreased and negatively correlated with circFADS2 expression in NSCLC tissues. CircFADS2 inhibition significantly increased miR-498 expression in lung cancer cells. Furthermore, we showed that miR-498 inhibitors reversed the function of circFADS2 on NSCLC cells proliferation and invasion.

In conclusion, our findings showed that circFADS2 expression was up-regulated and associated with poor overall survival in NSCLC patients. Furthermore, we showed that circFADS2 promoted NSCLC progression by sponge of miR-498. Our study indicated that circFADS2 could be a potential therapeutic target for lung cancer treatment.

## Abbreviations

CCK-8: Cell Counting Kit-8
DMEM: Dulbecco modified Eagle medium
NSCLC: non-small cell lung cancer
